# Methicillin-Resistant Staphylococcus aureus Septic Pulmonary Embolism Presumed to Originate From Anal Canal Cancer With a Cutaneous Fistula: A Case Report

**DOI:** 10.7759/cureus.104352

**Published:** 2026-02-27

**Authors:** Hiroaki Nishimura, Shota Kaburaki, Koichiro Kamio, Kazuo Kasahara, Masahiro Seike

**Affiliations:** 1 Department of Pulmonary Medicine and Oncology, Graduate School of Medicine, Nippon Medical School, Tokyo, JPN; 2 Department of Pulmonary Medicine, Saitama Medical Center, Saitama Medical University, Kawagoe, JPN

**Keywords:** anal canal cancer, bacteremia, cutaneous fistula, mrsa, septic pulmonary embolism

## Abstract

Septic pulmonary embolism (SPE) is a severe condition often linked to infective endocarditis (IE) or intravascular catheter infections, with *Staphylococcus aureus *being a common pathogen. However, SPE originating from a gastrointestinal malignancy, particularly when caused by a non-enteric pathogen like methicillin-resistant *Staphylococcus aureus *(MRSA) via a malignancy-associated fistula, is rarely reported. We report the case of a 49-year-old man undergoing preoperative chemotherapy for anal canal cancer who developed MRSA-induced SPE complicated by multiple lung abscesses. Initial investigations revealed multiple bilateral pulmonary nodules with cavitation on chest computed tomography, and blood and sputum cultures subsequently grew MRSA. No evidence of IE was found on transthoracic echocardiography, and other common sources of SPE were not identified. A cutaneous fistula adjacent to the anal canal cancer, without overt signs of local infection, suggested that the cutaneous fistula was a plausible portal of entry for MRSA. The patient was successfully treated with an extended course of anti-MRSA therapy, including linezolid, and supportive care, leading to clinical and radiological improvement. This case highlights that anal canal cancer with an associated cutaneous fistula may serve as an occult portal of entry for MRSA, leading to SPE. Clinicians should consider this atypical mechanism in patients with malignancy presenting with SPE, especially when common sources are absent. Prompt recognition and targeted antimicrobial therapy are crucial for managing such complex infections.

## Introduction

Septic pulmonary embolism (SPE) is a serious condition resulting from the hematogenous spread of infected thrombi to the pulmonary vasculature, typically originating from an extrapulmonary infection focus. While relatively uncommon, SPE can lead to significant morbidity and mortality if not diagnosed and treated promptly [1]. Clinical presentations often include fever, dyspnea, pleuritic chest pain, and cough, though symptoms can be non-specific, necessitating a high index of suspicion [1,2]. Characteristic computed tomography (CT) findings include multiple, often bilateral, peripheral pulmonary nodules, frequently with cavitation, wedge-shaped infiltrates, and pleural effusions [1,3-5]. The most common predisposing factors for SPE are intravenous drug use, indwelling medical catheters, and skin and soft tissue infections [1,2,6,7]. *Staphylococcus aureus*, including both methicillin-susceptible *Staphylococcus aureus* (MSSA) and methicillin-resistant *Staphylococcus aureus *(MRSA) strains, is the predominant pathogen isolated in patients with SPE [1,3]. Because symptoms are often non-specific and imaging findings may mimic pneumonia or metastatic disease, delayed recognition of SPE can occur and may adversely affect outcomes, particularly in immunocompromised hosts.

MRSA has emerged as a significant pathogen in SPE, accounting for a substantial proportion of *Staphylococcus aureus *isolates in various cohorts, particularly in healthcare-associated infections or in patients with specific risk factors such as indwelling catheters or prior antibiotic exposure [1,3]. While the clinical and radiological presentations of MRSA-induced SPE are largely indistinguishable from those caused by MSSA, the implications for treatment and outcomes are significant, underscoring the need for early and appropriate antimicrobial therapy targeting MRSA when suspected [2]. In parallel, the association between certain gastrointestinal (GI) malignancies, particularly colorectal cancer, and bloodstream infections with specific enteric bacteria is well-documented. Organisms such as *Streptococcus gallolyticus *(formerly *Streptococcus bovis*) [8] and *Clostridium septicum *[9] have been implicated in bacteremia that can unmask an underlying GI malignancy [10], sometimes leading to infective endocarditis (IE) and subsequent embolic phenomena. However, SPE directly originating from a GI malignancy itself, particularly when caused by non-enteric flora such as MRSA, and in the absence of classical predisposing factors like IE or central venous catheters, is a rarely reported clinical scenario.

Here, we present a case of SPE and multiple lung abscesses caused by MRSA in a patient with anal canal cancer and an associated cutaneous fistula, in the absence of IE or other common sources of SPE. This case highlights a potentially underrecognized mechanism wherein a cutaneous fistula adjacent to a malignancy can serve as a portal of entry for MRSA, leading to severe systemic infection, and underscores the importance of considering unusual infection sources in patients with malignancy and unexplained septic embolism.

## Case presentation

**Figure 1:**
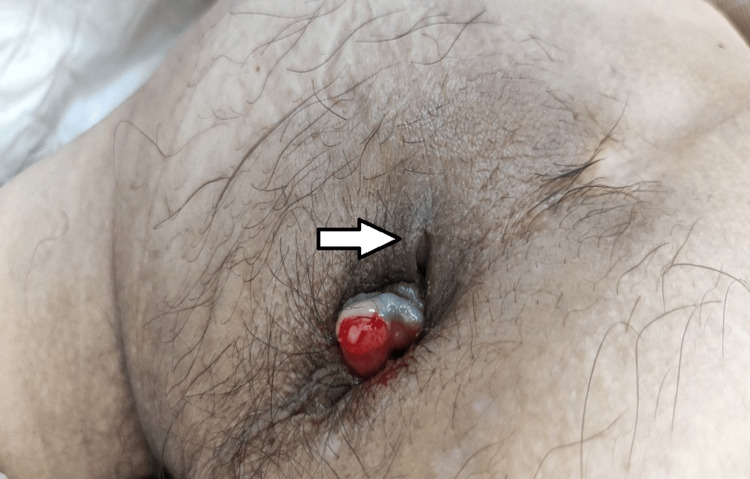
Perianal examination findings An exposed tumor mass is visible around the anus, with a cutaneous fistula noted in the vicinity (arrow). No overt signs of purulent discharge or significant inflammation were present around the fistula.

A 49-year-old man presented to our department with a chief complaint of lightheadedness that had developed approximately five days prior to his unscheduled visit. He was receiving preoperative chemotherapy, consisting of capecitabine and oxaliplatin, for stage IIIB anal canal cancer. He presented with cough and low-grade fever. His past medical history was notable for anal polyps diagnosed at age 13 and colon polyps at age 34. He had no other significant comorbidities, and his current immunocompromised state was considered secondary to his underlying malignancy and ongoing chemotherapy. He had never smoked and reported no history of alcohol consumption. Prior to this admission, his medications included magnesium oxide and oxycodone for anal pain. His vital signs were as follows: heart rate 115 beats per minute, respiratory rate 19 breaths per minute, blood pressure 122/79 mmHg, and oxygen saturation 94% while breathing ambient air. Physical examination revealed coarse crackles in both lungs, with diminished breath sounds in the right lower lung field. The abdomen was flat and soft, without tenderness. Examination of the perianal region revealed an exposed tumor mass with an adjacent cutaneous fistula (Figure 1).

Anal pain was present, but notably, cardinal signs of inflammation, including erythema, edema, purulent discharge, fluctuance, or local heat, were absent in the perianal region surrounding the fistula. Initial laboratory investigations revealed a white blood cell count of 16,500/μL (neutrophils 15,659/μL), hemoglobin of 9.5 g/dL, and a platelet count of 142,000/μL. Inflammatory markers were significantly elevated, with a C-reactive protein level of 27.5 mg/dL and a procalcitonin level of 90 ng/mL. D-dimer was also elevated at 5.3 μg/mL. Serum (1→3)-β-D-glucan was within normal limits at 12 pg/mL (Table 1).

**Table 1:** Blood test on admission WBC: white blood cell count; RBC: red blood cell count; Hb: hemoglobin; MCV: mean corpuscular volume; MCH: mean corpuscular hemoglobin; MCHC: mean corpuscular hemoglobin concentration; Plt: platelet count; TP: total protein; Alb: albumin; BUN: blood urea nitrogen; Cr: creatinine; CRP: C-reactive protein

Variable	Reference range, adults	On admission
Hematology		
WBC	3,900-8,800/µL	16,500/µL
RBC	380-560×10⁴/µL	411×10⁴/µL
Hb	13.4-17.5 g/dL	9.5 g/dL
MCV	83.8-103.6 fL	90.9 fL
MCH	28.3-35.3 pg	30.7 pg
MCHC	31.6-35.9%	33.8%
Plt	13.9-37.3×10⁴/µL	14.2×10⁴/µL
Biochemistry		
TP	6.6-8.1 g/dL	6.1 g/dL
Alb	4.1-5.1 g/dL	2.1 g/dL
BUN	8.0-20.0 mg/dL	13.7 mg/dL
Cr	0.65-1.07 mg/dL	0.50 mg/dL
Inflammatory and serological markers		
CRP	<0.30 mg/dL	27.50 mg/dL
Procalcitonin	<0.05 ng/mL	90.00 ng/mL
(1→3)-β-D-glucan	<11.0 pg/mL	12.2 pg/mL

**Figure 2:**
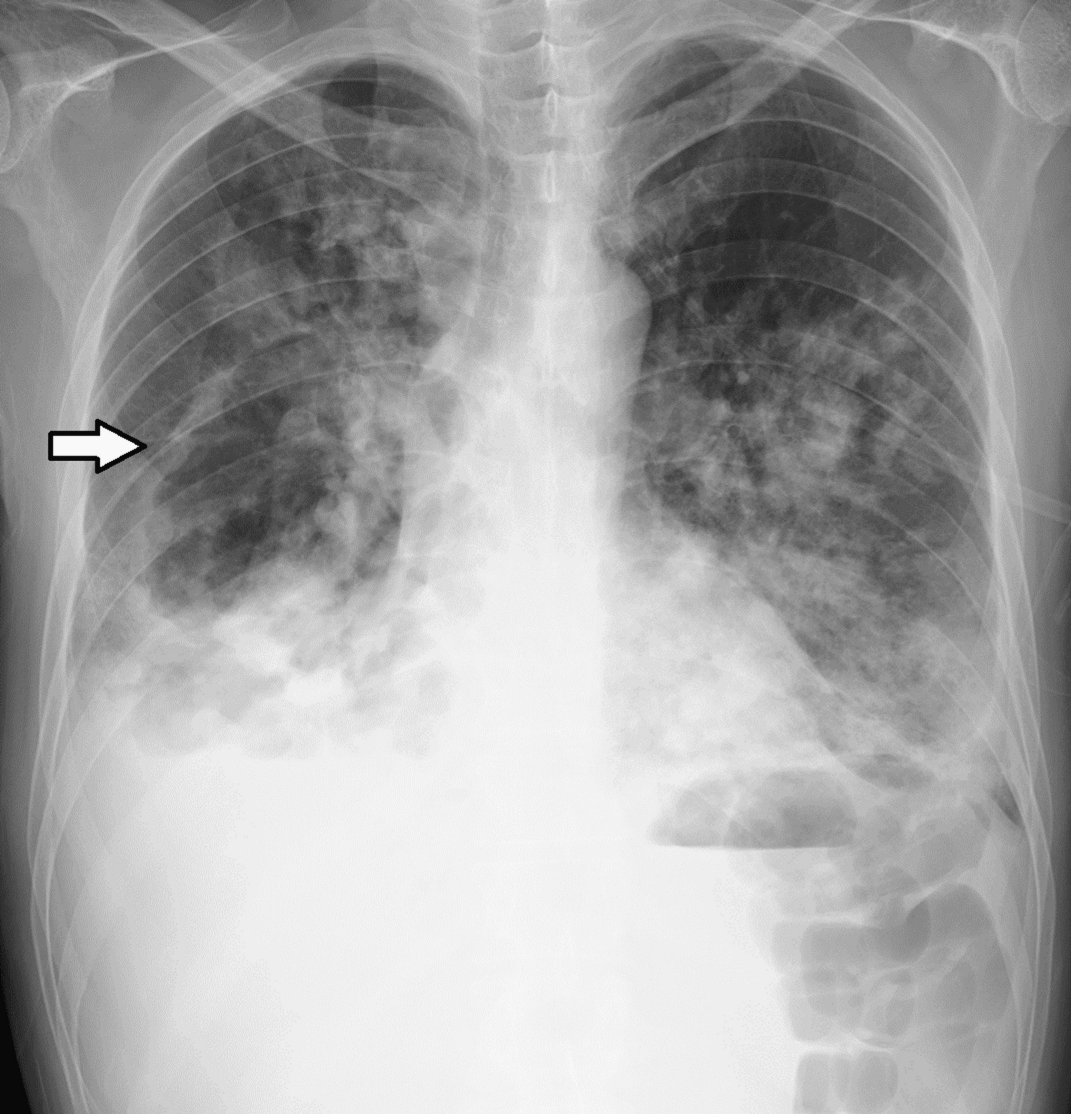
Chest radiograph on admission The radiograph shows a cavitary shadow in the right middle lung field (arrowhead) and diffuse infiltrative shadows in the right upper lung field as well as the left middle and lower lung fields.

Given the patient's immunocompromised state due to chemotherapy and the presence of fever, a severe infectious etiology was suspected. Considering the elevated inflammatory markers and the possibility of bacteremia/sepsis, two sets of blood cultures and sputum cultures were obtained prior to antibiotic administration; these cultures subsequently grew MRSA. Furthermore, as the initial chest CT revealed cavitary lung lesions, sputum acid-fast bacilli (AFB) smears and cultures were performed as part of the differential diagnostic workup to exclude pulmonary tuberculosis. The isolated MRSA demonstrated sensitivity to linezolid and vancomycin (VCM). Three sputum smears for AFB were negative, as were sputum cultures for mycobacteria and polymerase chain reaction assays for *Mycobacterium tuberculosis* and *Mycobacterium avium complex*. Serological tests for hepatitis B surface antigen, hepatitis C virus antibody, and human immunodeficiency virus antibody and antigen were negative. An interferon-gamma release assay for tuberculosis yielded a negative result. Rapid antigen tests for COVID-19 and influenza, as well as urinary antigen tests for *Streptococcus pneumoniae* and *Legionella pneumophila*, were all negative. Fungal markers, including *Aspergillus *antigen, *Candida* mannan antigen, and cryptococcal antigen, were also negative. An oral examination performed by a dentist ruled out odontogenic infections, such as periodontal disease or dental abscesses. A chest X-ray demonstrated a cavitary shadow in the right middle lung field, along with infiltrative shadows in the right upper lung field and the left middle and lower lung fields (Figure 2).

**Figure 3:**
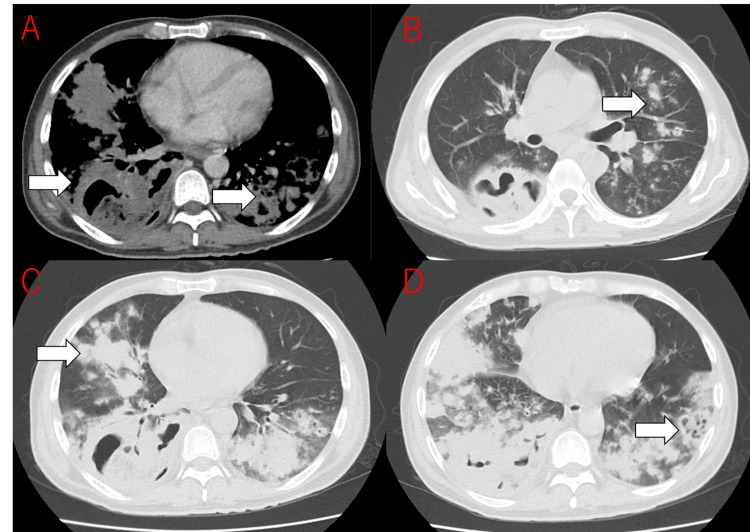
Chest computed tomography scan on admission Axial (A, B, C, D) views demonstrate multiple, bilateral, peripheral nodular infiltrates (arrows) and a distinct cavitary lesion in the lower lobe of the right lung, consistent with septic pulmonary emboli.

**Figure 4:**
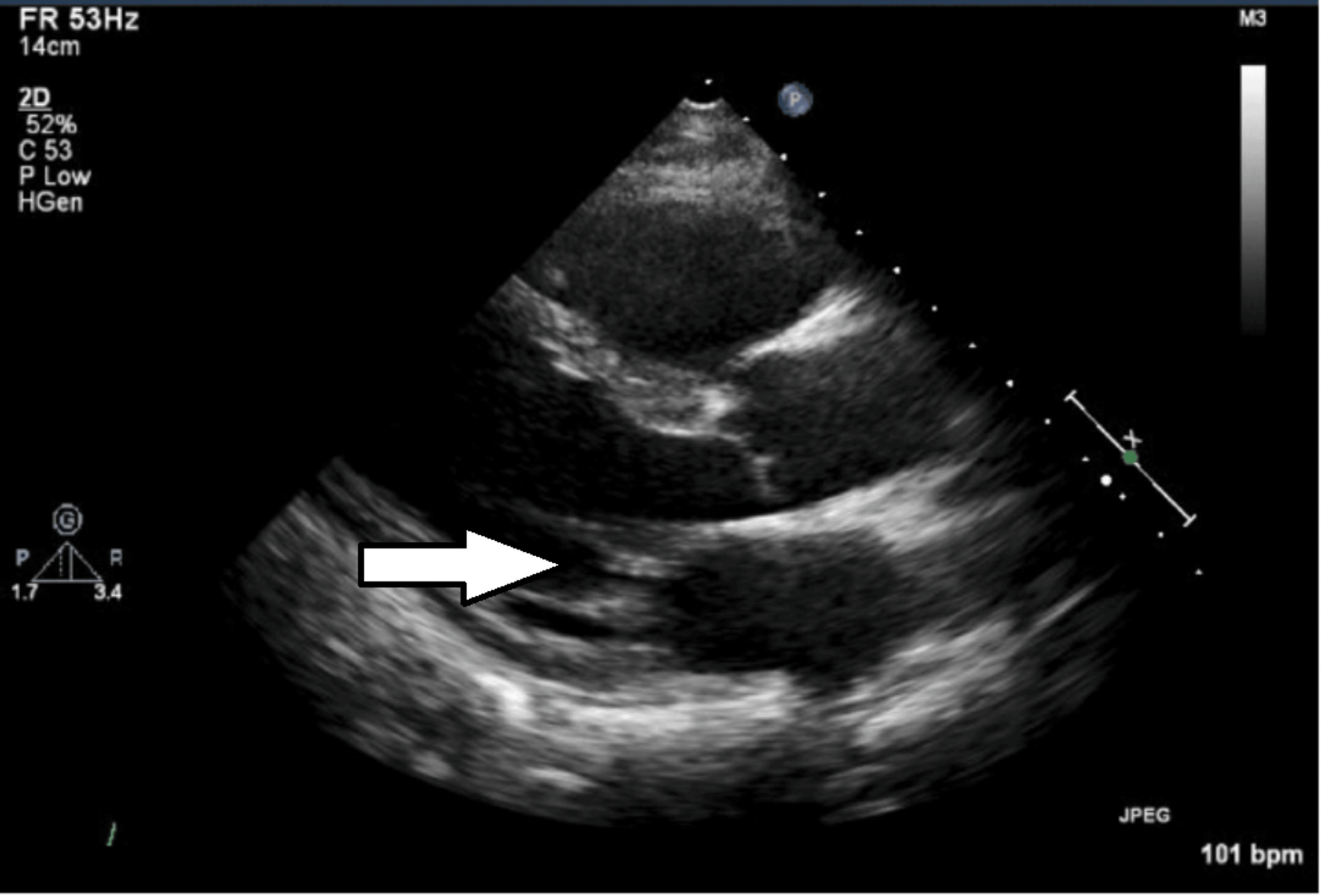
Transthoracic echocardiogram Parasternal long-axis view shows mild thickening of the mitral valve leaflets (arrows). No obvious vegetations or significant valvular regurgitation suggestive of infective endocarditis were observed.

A subsequent chest CT scan revealed multiple scattered infiltrative and nodular shadows throughout both lungs, with a distinct cavitary lesion identified in the lower lobe of the right lung (Figure 3). Transthoracic echocardiography (TTE) showed mild thickening of the mitral valve leaflets but no obvious vegetations or significant valvular regurgitation to suggest IE (Figure 4). On hospital day 2, empiric antimicrobial therapy with piperacillin/tazobactam (PIPC/TAZ) was initiated due to suspected severe infection. Blood cultures drawn on admission subsequently revealed staphylococcal bacteremia on day 3, prompting the addition of VCM. VCM dosage was managed using area under the concentration-time curve area under the curve (AUC)-guided therapeutic drug monitoring to ensure efficacy and safety. By day 4, both blood and sputum cultures confirmed MRSA, leading to the discontinuation of PIPC/TAZ. Based on these microbiological findings, along with the clinical presentation and characteristic chest CT findings of multiple bilateral nodules with cavitation, a diagnosis of SPE and multiple lung abscesses due to MRSA was established. IE was considered unlikely given the echocardiographic findings.

**Figure 5:**
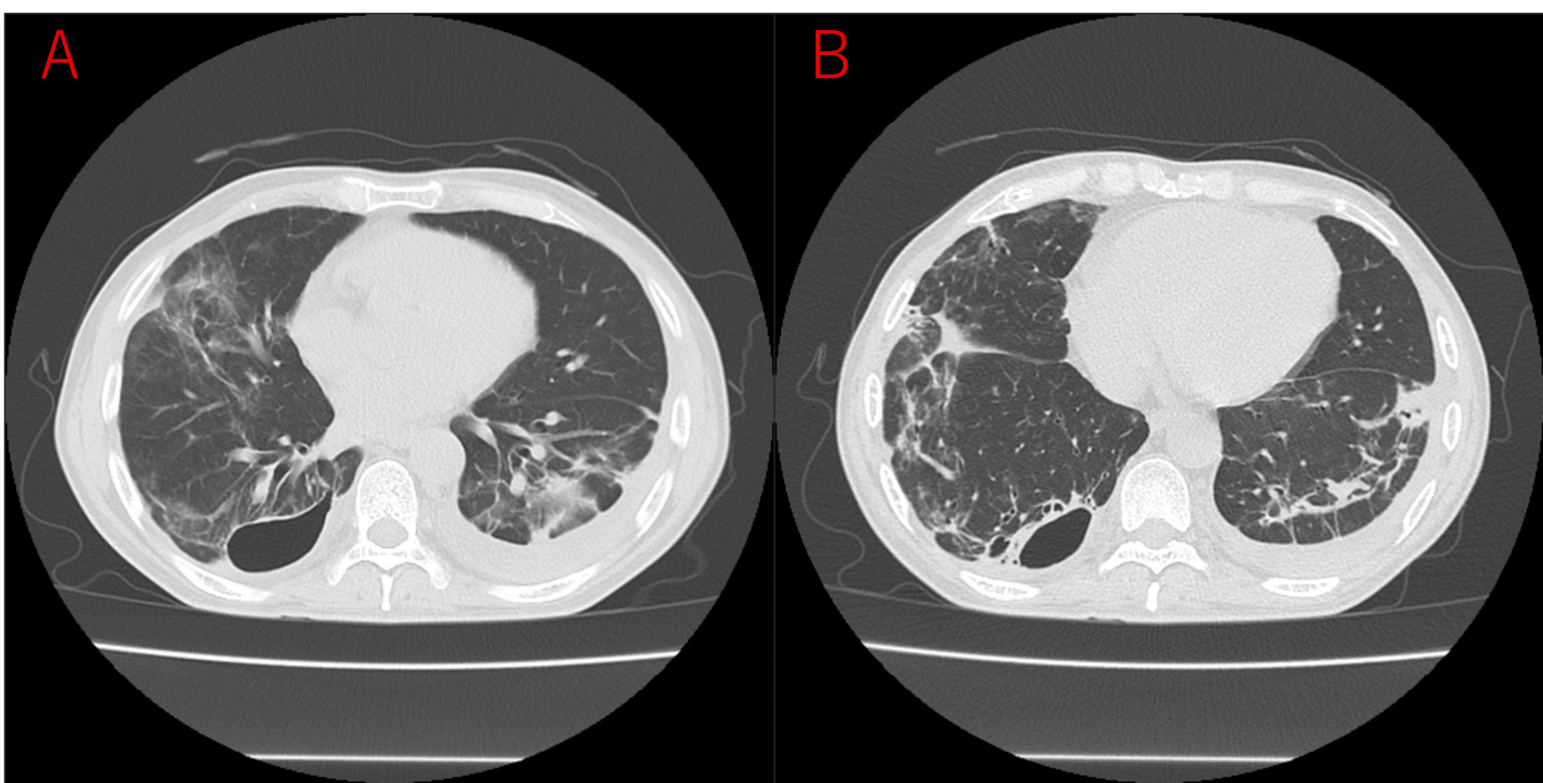
Follow-up chest computed tomography scan at discharge Axial (A, B) view shows considerable resolution of the previously observed pulmonary infiltrates and cavitary lesions in the right lung compared to the admission computed tomography (Figure 3).

The anal canal cancer with its associated cutaneous fistula was suspected as the primary portal of entry for the MRSA bacteremia, as no other definitive source of infection was identified. The patient responded to the VCM therapy, and blood cultures became negative for MRSA by day 8. Sputum cultures were negative by day 21. On day 29, a diagnostic thoracentesis performed for a left-sided pleural effusion yielded pleural fluid that was culture-negative for MRSA. Considering the superior tissue penetration of linezolid into lung parenchyma and the feasibility of oral administration to prioritize the management of his underlying anal canal cancer and facilitate discharge, the antimicrobial regimen was transitioned to oral linezolid on day 31. The patient responded well to the extended course of antimicrobial therapy. By day 53 of hospitalization, his inflammatory markers had normalized, and there was a marked improvement in both his clinical symptoms and the radiological findings on chest imaging. Consequently, he was discharged home on day 57 to continue oral linezolid and to prepare for the subsequent management of his anal canal cancer. A follow-up chest CT scan at the time of discharge demonstrated considerable resolution of the previously observed pulmonary infiltrates and cavitary lesions (Figure 5).

The detailed clinical course, including the timing of antimicrobial adjustments, microbiological findings, and the patient's response to treatment, is summarized in the Appendices.

## Discussion

We describe a case of SPE and multiple lung abscesses due to MRSA in a patient undergoing chemotherapy for anal canal cancer. The notable aspects of this case include, first, the occurrence of MRSA-induced SPE where common sources like IE or central venous catheter-related bloodstream infection were absent and, second, the clinical evidence suggesting that a cutaneous fistula associated with the anal canal cancer may have provided a portal of entry for MRSA, leading to hematogenous spread. These observations highlight a less commonly reported mechanism for SPE, involving a non-enteric pathogen potentially originating from a malignancy-associated fistula.

SPE commonly arises from IE, particularly right-sided, or infections associated with intravascular devices such as central venous catheters [1,2]. *Staphylococcus aureus*, including MRSA, is a frequently implicated pathogen in these scenarios [1,3]. In our patient, TTE provided adequate visualization and did not reveal vegetations suggestive of IE. Given the absence of echocardiographic evidence of endocarditis on TTE and the patient's rapid clinical improvement with appropriate antimicrobial therapy, transesophageal echocardiography (TEE) was not pursued to avoid additional invasiveness. Compared to TTE, TEE offers significantly higher sensitivity for detecting small vegetations (often <5 mm) and assessing valvular structures due to the higher frequency of the transducer and its closer proximity to the heart without interference from the chest wall or lungs. This makes TEE the preferred modality when clinical suspicion of IE remains high despite a negative TTE [11]. While skin and soft tissue infections are also recognized sources of SPE [2,3], the absence of overt inflammation or purulent discharge around the perianal tumor and fistula at presentation made a typical skin or soft tissue abscess less likely as the direct source, although microscopic invasion cannot be entirely excluded. Therefore, the occurrence of MRSA-induced SPE in this context, without a clearly identifiable common predisposing factor, prompted a search for an alternative portal of entry. Although primary MRSA necrotizing pneumonia with secondary bacteremia was considered in the differential diagnosis, the clinical course lacked preceding severe respiratory viral symptoms often seen in primary pneumonia. Furthermore, the characteristic chest CT findings, specifically the multiple, bilateral, peripheral nodular infiltrates with cavitation, strongly supported a diagnosis of SPE resulting from hematogenous spread rather than primary respiratory infection [1,4].

The association between certain GI malignancies and bloodstream infections is recognized, most notably the link between colorectal cancer and bacteremia with enteric organisms such as *Streptococcus gallolyticus *or *Clostridium septicum*, which can lead to complications like endocarditis or metastatic infection [9,12]. However, MRSA is not a typical component of the gut flora [13]. In our case, the presence of an anal canal cancer with an adjacent cutaneous fistula, which showed no overt signs of active infection like purulent discharge or significant surrounding inflammation, is suspected to have served as an occult portal of entry for MRSA. It is plausible that MRSA, colonizing the perineal skin, gained access to the systemic circulation via the compromised tissue integrity within the fistulous tract and the underlying tumor microenvironment. This mechanism, involving translocation of skin flora through a malignancy-associated fistula, mirrors pathways described in SPE arising from other types of fistulas or chronic soft tissue infections, where disruption of anatomical barriers allows bacterial invasion [14,15]. While direct invasion of MRSA from an ulcerated tumor surface is also a consideration, the fistula provides a more distinct anatomical conduit from the skin.

The clinical implication of this case is that in patients with malignancy, particularly those involving anatomical sites prone to fistula formation or communication with the skin surface like anal canal cancer, clinicians should maintain a degree of suspicion for SPE even when typical sources are absent and the causative pathogen is not classically associated with enteric infections. The presence of a fistula, even if appearing uninfected clinically, may represent a compromised barrier allowing for microbial translocation. This is particularly relevant for immunocompromised patients, such as those undergoing chemotherapy, where susceptibility to infection from skin flora might be increased. Furthermore, this case suggests that MRSA should be considered a potential pathogen in tumor-associated bacteremia and subsequent SPE if a plausible link to the skin or an external communication exists, prompting thorough evaluation for such anatomical abnormalities. Early recognition of these atypical presentations and consideration of broader differential diagnoses for infection sources in cancer patients could facilitate timely and targeted antimicrobial therapy, potentially improving outcomes as highlighted by studies emphasizing the importance of prompt, effective treatment for SPE [2].

We acknowledge several limitations in this report. First, a microbial culture directly from the cutaneous fistula was not obtained prior to antimicrobial administration. This was primarily because the fistula appeared dry without overt purulent discharge or exudate at the time of presentation, limiting the feasibility of obtaining an adequate sample. Microbiological confirmation of the local flora matching the blood isolates would have provided stronger evidence to support our hypothesis. Second, cross-sectional imaging of the pelvis (such as enhanced CT or magnetic resonance imaging (MRI)) was not performed, which limited our ability to fully evaluate the depth of the fistula tract or the presence of a deep-seated abscess. However, the absence of other identifiable sources, including the lack of evidence of IE and odontogenic infections, suggests that the fistula was a plausible portal of entry.

## Conclusions

This case report describes an instance of MRSA-induced SPE where an anal canal cancer with an associated cutaneous fistula was the presumed portal of entry. While a direct causal link cannot be definitively proven in a single case report, the clinical evidence suggests this unusual mechanism. This case serves as a reminder that clinicians should consider the possibility of non-enteric pathogens, such as MRSA, causing SPE in patients with malignancies, especially when anatomical abnormalities like fistulas provide a potential conduit for skin flora to enter the systemic circulation. A high index of suspicion for atypical sources of infection is warranted in immunocompromised patients with unexplained septic conditions, and thorough investigation for underlying anatomical defects should be a part of the diagnostic approach.

## Appendices

**Table 2:** Clinical course and key milestones SPE: septic pulmonary embolism; CT: computed tomography; MRSA: methicillin-resistant *Staphylococcus aureus*

Hospital day	Key events/management	Microbiology	Imaging/clinical response
1 (admission)	Fever/cough; SPE suspected; blood and sputum cultures obtained	Pending	CT: multiple bilateral peripheral nodules with cavitation
2	Started empiric piperacillin/tazobactam	-	-
3	Staphylococcal bacteremia reported; added vancomycin	Blood culture: staphylococci	-
4	MRSA confirmed; stopped piperacillin/tazobactam	Blood and sputum: MRSA	-
8	-	Blood cultures became negative	Clinical improvement
21	-	Sputum cultures became negative	-
29	Thoracentesis for pleural effusion	Pleural fluid culture: negative	-
31	Switched to oral linezolid	-	-
53	Continued therapy	-	Inflammatory markers normalized; marked radiologic improvement
57 (discharge)	Discharged with oral linezolid	-	Follow-up CT: considerable resolution
